# Stu2 is required for plus-end directed chromosome transport along microtubules during metaphase in *Saccharomyces cerevisiae*

**DOI:** 10.17912/micropub.biology.001577

**Published:** 2025-04-28

**Authors:** Julia R Torvi, Jonathan Wong, David G Drubin, Georjana Barnes

**Affiliations:** 1 Department of Molecular and Cell Biology, University of California, Berkeley, Berkeley, California, United States; 2 Biophysics Graduate Group, University of California, Berkeley, Berkeley, California, United States

## Abstract

Chromosome alignment on the mitotic spindle, also referred to as congression, is facilitated by translocation of side-bound chromosomes along the microtubule surface, which allows the establishment of end-on attachment of kinetochores to microtubule plus ends. We use a reconstitution assay in lysates prepared from metaphase-arrested budding yeast to show that kinetochore translocation along the lateral surface of microtubules is dependent on Stu2, a homolog of vertebrate XMAP215. Stu2 tracks both growing and shrinking microtubule ends but also colocalizes with moving lattice-bound kinetochores. In cells, we observed that Stu2 depletion impairs chromosome biorientation during metaphase.

**Figure 1. Stu2 is required for lateral kinetochore movement on metaphase microtubules:**
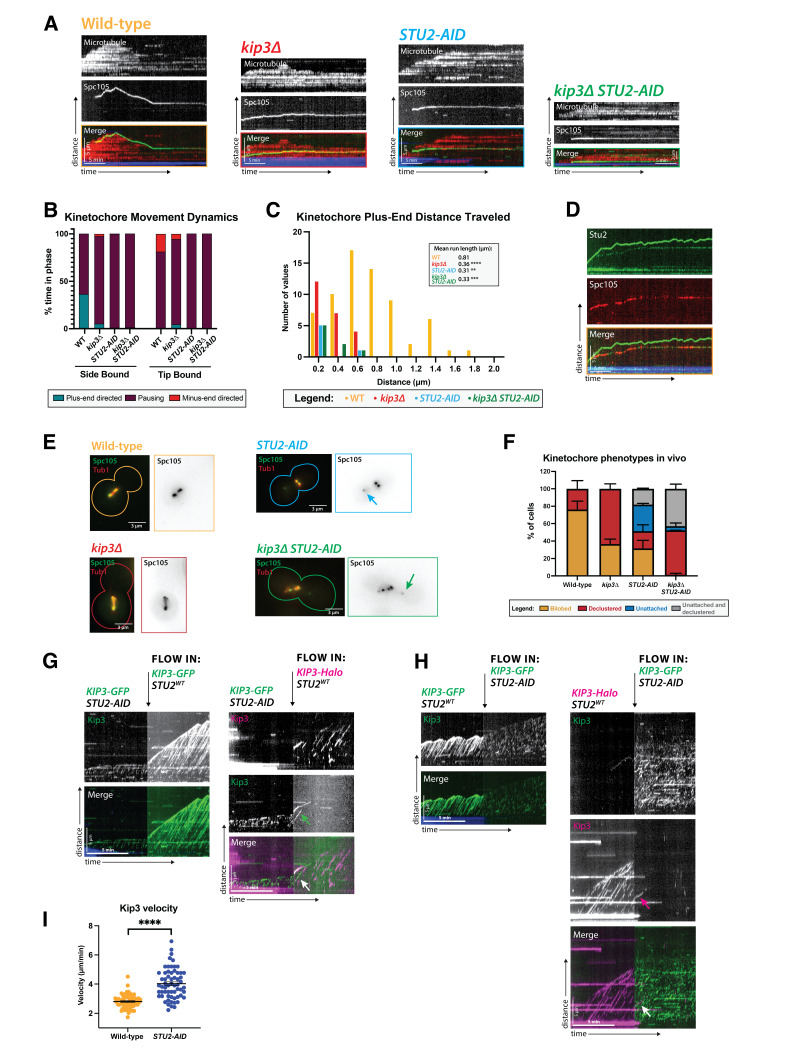
(A) Kinetochore movement toward the microtubule plus end is dependent on Stu2, similar to its Kip3-dependence. Representative kymographs from metaphase-arrested lysates made from cells expressing Spc105-GFP, an outer kinetochore protein. Time is on the x-axis, and distance is on the y-axis (scale bars are 5 min and 5 µm, respectively). The kinetochore (Spc105-GFP) is green, the yeast microtubule (mRuby2-Tub1) is red, and the microtubule seed is blue. In wild-type lysates, kinetochores move toward the microtubule plus end when bound to its lateral surface and track the microtubule tip after becoming end-bound. (B) Quantification of kinetochore dynamics over time shows they spend 36.16% of their time moving toward the plus end of the microtubule when laterally bound to the microtubule. This value decreases to 5.18%, 1.13%, and 1% in *kip3∆,* *STU2-AID,* and *kip3∆ STU2-AID* lysates, respectively. When a kinetochore reaches the plus end of a microtubule, it remains bound to the tip and can move away from the seed (plus-end directed), toward the seed (minus-end directed), or remain paused if the microtubule polymerizes, depolymerizes, or is static, respectively. In lysates from mutant strains, reduced minus-end directed movement of tip-bound kinetochores is observed compared to wild-type. (C) Both run lengths and the number of runs the kinetochores make toward the microtubule plus ends depend on Kip3 and Stu2. While the wild-type run length was 0.81 µm, in *kip3∆,* *STU2-AID,* and *kip3∆ STU2-AID* lysates, run lengths were reduced to 0.36 µm, 0.31 µm, and 0.33 µm, respectively. Statistical analysis was done by a Kruskal-Wallis test, wherein **** is p <0.0001, *** is p = 0.0005, and ** is p = 0.0021. (D) Representative kymograph showing wild-type metaphase arrested lysate with Stu2-GFP (green) colocalizing with Spc105-mScarlet-I (red) on the microtubule lattice. The microtubule seed is shown in blue. Stu2 at the plus end of the microtubule is brighter and distal from the seed, while Stu2 on the side of the microtubule is dimmer and more proximal to the seed. 97% (56/58) of lattice-bound kinetochore tracks overlapped with Stu2 tracks. Time is on the x-axis, and distance is on the y-axis (scale bars are 5 min and 5 µm, respectively). (E) Stu2 depletion interferes with chromosome clustering and attachment in metaphase cells, similar to *kip3∆* mutants. Metaphase cells with wild-type, *kip3* Δ, *STU2-AID,* or *kip3∆ & STU2-AID* alleles were imaged in a *cdc23-1* strain expressing Spc105-GFP (green) and mRuby2-Tub1 (red). Note that these cells were not shifted to non-permissive temperature and were not arrested in the cell cycle for imaging because the bud morphology indicated the cell cycle stage. Each image shown is a maximum Z-projection of a 5 µm stack of 0.2 µm slices. (F) Quantification of the declustered and unattached kinetochore phenotype is presented as a bar graph for spindles that are 2–3 µm in length. Cells were classified as ‘bilobed’ by a line scan showing two distinct peaks (represented in the wild-type image). The ‘declustered’ phenotype included all line scans that did not show two distinct peaks (represented in the *kip3∆* image). The ‘unattached’ phenotype was classified as the presence of at least one kinetochore signal off-axis away from the main spindle (represented in the *STU2-AID* image). Two biological replicates were analyzed, each with n=50 cells. Error bars are standard errors of the mean. (G) Stu2 increases Kip3 processivity in metaphase yeast lysate. Kip3-GFP was imaged in a *STU2-AID* lysate, showing short non-processive motor tracks. Lysate containing wild-type Stu2 was added to the flow chambers, and the Kip3 processivity was tracked either with Kip3-GFP (left) or with far red Kip3-Halo (right). Arrows show areas in which Kip3 from the *STU2-AID* strain had its processivity restored once wild-type Stu2 was added. (H) Stu2 depletion decreases Kip3 processivity in metaphase yeast lysate. Kip3-GFP (left) or far red Kip3-Halo (right) was imaged in wild-type *STU2* lysate showing long processive motor tracks. Lysate from *STU2-AID* strains, depleted for Stu2, replaced the wild-type lysate in the flow chambers, and the Kip3 processivity was tracked with Kip3-GFP. Arrows show areas in which Kip3 from the wild-type *STU2* strain lost processivity once *STU2-AID* lysate replaced the wild-type lysate. (I) Kip3 velocities were quantified revealing that Kip3 moves faster upon Stu2 depletion, showing an increase in velocity from 2.8 µm/min to 4.0 µm/min. The SEM is shown in black. Statistical analysis was done using a Welch's t test where **** is p<0.0001. Quantification is from two replicate trials. N equals 57 and 61 Kip3 proteins tracked for lysate from wild-type and *STU2-AID* strains, respectively.

## Description

Mitosis is an essential process by which replicated chromosomes are segregated to daughter cells. To achieve this, chromosomes are physically partitioned into daughter cells via a microtubule-mediated process. The process by which chromosomes align at the center of the mitotic spindle during metaphase for proper partitioning is called chromosome congression (Maiato et al., 2017). Chromosome congression is a complex process dependent upon motor forces (Cottingham et al., 1999), spindle geometry (Risteski et al., 2021), post-translational modifications (Barisic and Maiato, 2016), and chemical gradients (Heald and Khodjakov, 2015). In vertebrate cells, chromosomes that do not make end-on, plus end, attachment to kinetochore-microtubules can be transported to the plus end by a kinesin-7, CENP-E (Craske and Welburn, 2020; Iemura and Tanaka, 2015). Our previous research provided evidence that a kinesin-8, Kip3, may have a similar function in budding yeast (Torvi et al., 2022).

Another candidate protein for chromosome transport is the XMAP215 homolog, Stu2. In addition to its microtubule polymerase function, Stu2 has been shown to have a role at the kinetochore. Through immune-purifications, binding assays, cross-linking mass spectrometry, and crystallography, Stu2 has been shown to bind to kinetochores via interaction with the Ndc80 complex and aid in the establishment of proper kinetochore-microtubule attachments (Miller et al., 2016, 2019; Zahm et al., 2021). Furthermore, split fluorescent protein assays, yeast two-hybrid screens, and immune purifications indicate that Kip3 and Stu2 interact physically (Gandhi et al., 2011). Here, we show that Stu2 is necessary for plus-end directed kinetochore movement along microtubules in lysate from metaphase yeast and for chromosome clustering in vivo.

Using a previously described reconstitution assay that uses cell-cycle arrested whole cell lysates, we can observe dynamic kinetochores and MAPs on microtubules via TIRF microscopy (Bergman et al., 2018; Torvi et al., 2022). Kinetochore proteins (Spc105) and MAPs (Kip3, Stu2) were expressed at endogenous levels as fusions to fluorescent proteins in a strain background with fluorescently tagged alpha-tubulin (Tub1) and a temperature sensitive mutant ( *cdc23-1* ) that causes cells to arrest in metaphase at the non-permissive temperature (Irniger et al., 1995) In wild-type lysates, kinetochores (tracked by imaging Spc105-GFP) bind to the lateral surface of microtubules and move in a processive, directional manner toward the microtubule plus end, exhibiting an average run length of 0.81±0.059 µm ( Figure 1A- C). When bound to the lattice, the kinetochores remain mostly stationary, but spend approximately 36% of the time moving toward the plus end, and less than 1% of the time moving toward the minus end ( Figure 1B ). Due to both kinetochore movement toward the plus end and microtubule plus end dynamic assembly and disassembly, kinetochores eventually reach microtubule plus ends and establish end-on attachments. Here, we found that the microtubule polymerase, Stu2, is necessary for the directional and processive movement of lattice-bound kinetochores toward the plus end of microtubules. Its depletion reduces lateral kinetochore movement similarly to that observed in a *kip3∆* mutant ( Figure 1A- B). In the three mutant lysates, *kip3∆, STU2-AID,* and *kip3∆ STU2-AID* , the kinetochore run lengths decreased to 0.36±0.28, 0.31±0.040, and 0.33±0.045 µm, respectively ( Figure 1C ). To determine if Stu2 directly associates with lattice-bound kinetochores, we imaged Stu2-GFP in combination with a fluorescently tagged kinetochore protein, Spc105-mScarlet-I, in lysates derived from cells arrested at metaphase. Stu2 colocalized with 97% (56/58) of Spc105 observed on the lattice of microtubules, in addition to the dynamic plus ends ( Figure 1D ).

Although the reconstitution experiments described above make possible determining the behavior of different proteins on individual yeast microtubules in a cell-cycle specific manner, in the mitotic spindle, microtubule geometry and spindle forces are expected to influence protein behaviors (Trupinić et al., 2022). Therefore, we tested how Stu2 affects kinetochore biorientation within live cells and compared these phenotypes to *KIP3* mutants that likewise interfere with lateral kinetochore movement (Torvi et al., 2022). While Stu2 depletion in our assay resulted in immobile kinetochores, *STU2* null mutants also exhibit a short mitotic spindle in vivo (Kosco et al., 2001). The frequency of unattached kinetochores increased in the absence of Stu2, as reflected in the appearance of kinetochore spots far off-axis from the spindle, in agreement with previous reports (Kosco et al., 2001; Pearson et al., 2003; Severin et al., 2001). Additionally, these cells often display a declustered kinetochore phenotype (more than two kinetochore puncta on axis with the microtubules) similar to that of a *kip3∆* strain (Wargacki et al., 2010)( Figure 1E- F). These results are consistent with a model in which Stu2 has a role in chromosome congression, but a more precise disruption of Stu2's various functions would be required to isolate this mitotic effect from Stu2's other kinetochore roles, including microtubule nucleation at kinetochores to facilitate linkage to the spindle, sustaining lateral kinetochore attachment to microtubules, and regulation of kinetochore-microtubule attachment stability in response to tension (Abouelghar et al., 2024; Gandhi et al., 2011; Kitamura et al., 2010; Miller et al., 2016).

Since mutant *STU2* and *KIP3* alleles show similar defects in kinetochore movement along microtubules and with chromosome clustering, we tested whether perturbing either protein impacts the behavior of the other. While the localization of Stu2 in metaphase yeast lysates remained unchanged in *kip3∆* strains, the dynamics of Kip3 were altered in *STU2-AID* lysates. Instead of continuous runs at a velocity of 2.8±0.064 µm/min, the runs became very short and increased in velocity to 4.0±0.13 µm/min in the absence of Stu2 ( Figure 1G- I). We also demonstrated that adding wild-type *STU2* lysate to a *STU2-AID* lysate restored Kip3 processivity ( Figure 1G ). When this sequence was reversed, and the wild-type *STU2* lysate was replaced with *STU2-AID* lysate, Kip3 processivity was instantaneously diminished ( Figure 1H ). These results indicate that the absence of Stu2 affects more than just kinetochore motility along microtubules in this lysate assay. While kinetochores are immobile in a Stu2-depleted lysate, this could be due to either its role in mediating the interaction between Kip3 and the kinetochore or its impact on Kip3 motility, which subsequently results in the loss of kinetochore motility.

Here we demonstrate through an in vitro lysate assay that Stu2 is necessary for lattice-bound kinetochores to move toward the plus end of metaphase microtubules. Our evidence suggests that Stu2’s role in lateral kinetochore movement may be more complex than merely linking kinesin-8 to the kinetochore. Although Kip3 does not stably colocalize with lattice-bound kinetochores as Stu2 does (Torvi et al., 2022), it is possible that the interactions with the kinesin are too weak for detection by TIRF microscopy or too transient to be captured within the frame rate of our recordings. The effect of Stu2 on Kip3’s processivity adds another potential dimension in which its depletion could impede kinetochore transport. While further studies are essential to elucidate the precise molecular mechanisms of Kip3 and Stu2 clearly, our data offer new insights into their roles in chromosome congression.

## Methods

Yeast strains, culturing, and harvesting

Yeast strains used in this study are listed below. Fluorescent protein fusion tags, degradation tags, and deletion constructs were designed and inserted as described previously (Bindels et al., 2017; Lee et al., 2013; Longtine et al., 1998). Strains were grown in yeast extract peptone dextrose (YPD) rich medium at 25ºC because they contain a temperature-sensitive allele.

Cells were harvested as described previously (Torvi et al., 2022). To specifically degrade a protein of interest, strains containing a degron tag were arrested at 30ºC for 3 hours (total) and then treated with 250 µM 3-indole acetic acid (Sigma-Alrich, St. Louis, MO) for 30 minutes before harvesting.

Generation of whole cell lysates

Approximately 5g of frozen cells were weighed into a pre-chilled medium-sized SPEX 6870 freezer mill vial (Spex, Metuchen, NJ). The chamber was then cryo-milled as follows: 3-minute pre-chill, 10 cycles of 3 minutes grinding at 30 impacts per second (15 cps), with 1 minute of rest between grinds. The resulting powered lysate was stored at -80ºC.

Assembly of flow chamber with stabilized, biotinylated, far-red labeled tubulin seeds

The flow chamber for use in the TIRF-based dynamics assay was prepared as previously described (Torvi et al., 2022).

Preparation of cell lysates for dynamics assay

Similar to what was previously described (Torvi et al., 2022), a 1.5 mL Eppendorf tube was pre-chilled in liquid nitrogen and filled with 0.22 g of powdered lysate. A microtubule length of ~ 5 µm is optimal for visualization. To create microtubules of this length, varying volumes of cold 10X PEM (800 mM PIPES pH 6.9, 10 mM MgCl2, 10 mM EGTA) were added to the lysate. Different strains required different buffer volumes to achieve the desired length in the assay (between 2-15 µL). The lysate was then combined with 0.5 µL of Protease Inhibitor Cocktail IV (Calbiochem, San Diego, CA) and 4 µL of 25.5 U/µL benzonase nuclease (EMD Millipore, San Diego, CA, prod. 70746-3, >90% purity) and thawed on ice for 10 minutes. The thawed lysate was then transferred to pre-chilled polycarbonate ultracentrifuge tubes and cleared of insoluble material by spinning at 34,600 x g for 25 minutes at 4ºC. Finally, 32 µL of the cleared lysate supernatant was flowed into a flow chamber for imaging.

Sequential lysate flow assays

For sequential lysate imaging, 32 µL of the first lysate was flowed through the chamber and imaged for 7.5 minutes to observe initial behavior. While the slide was still mounted on the microscope, the movie was paused for 1 minute while the second lysate was carefully flowed in to replace the first. Once the lysate was replaced, the movie was restarted, and the same field of view was captured for another 7.5 minutes. Frames were captured every 2.5 seconds for a total of 15 minutes.

TIRF Microscopy

After adding the clarified lysate supernatant to the prepared chamber, the slides were loaded onto a Nikon Ti2-E inverted microscope with an Oko Labs environmental chamber pre-warmed to 28ºC. Images were collected using a Nikon 60X CFI Apo TIRF objective (NA 1.49) and an Orca Fusion Gen III sCMOS camera (Hamamatsu, Hamamatsu City, Japan) at 1.5X magnification using the Nikon NIS Elements software. A LUNF 4-line laser launch (Nikon Instruments, Melville, NY) and an iLas2 TIRF/FRAP module (Gataca Systems, Massy, France) were used to achieve total internal reflection fluorescence (TIRF), which illuminates the region proximal to the coverslip surface. Images were acquired every 5 seconds for 30 minutes unless otherwise noted.

In vivo live cell microscopy

Cells were grown overnight in YPD medium overnight at 25ºC. The next day, they were diluted into fresh YPD and cultured for approximately four hours (two doublings) at 25ºC until the culture reached log phase (OD600=0.5). The cells were then washed three times with Imaging Medium (synthetic minimal medium supplemented with 20 µg/ml adenine, uracil, L-histadine, and L-methionine; 30 µg/ml L-leucine and L-lysine; and 2% glucose; Sigma-Aldrich) and immobilized on coverslips coated with 0.2 mg/ml concanavalin A. Imaging was performed using a Nikon Ti2-E inverted microscope with an Oko Labs environmental chamber pre-warmed to 25ºC. Images were acquired using a Nikon 60X CFI Apo TIRF objective (NA 1.49) and an Orca Fusion Gen III sCMOS camera (Hamamatsu) at 1.5X magnification using Nikon NIS Elements software. A LUNF 4-line laser launch (Nikon) and an iLas2 TIRF/FRAP module (Gataca Systems) were used for HiLo total internal reflection fluorescence (Tokunaga et al., 2008). Images were taken with 0.2 µm slices for a total 5 µm Z-stack.

Image and data analysis

Imaging data were analyzed using Fiji software (NIH). Registration to correct for stage drift was applied (StackReg; Thévenaz et al., 1998). Kymographs were generated from all microtubules for which the entire length could be tracked for the entire movie. Kymographs were excluded if the microtubules were crossed or bundled. Data from independent technical trials and biological replicates from imaging of one strain were pooled, unless indicated otherwise. Kinetochore movements of less than a threshold slope of 2-pixel displacement (144.4 nm) per 10 pixels of time (50 sec) were categorized as “paused”. Kip3 velocities are reported as the mean with the standard error of the mean. Statistical significance was determined using a Kruskal-Wallis test for kinetochore travel distances and a Welch's t test for kinesin velocities (GraphPad Prism, San Diego, CA). P Values are reported as in the figure captions.

For the live cell imaging analysis, images were analyzed using Fiji (NIH). Maximum intensity Z-projections were made, and bleach corrections were applied, using the “histogram matching” macro. Metaphase cells were identified by the presence of a 2-3 µm spindle at the entrance to the bud neck. Cells were then counted as either “bilobed” or “declustered” based on the presence of two distinct kinetochore puncta. Line scans were performed to assist in this binary classification. If two clear peaks were present, the cell was classified as “bilobed.” Any other line scan shape, i.e., 3+ peaks or 1 long kinetochore signal that matched the spindle, was classified as “declustered.” Cells with kinetochores that were classified as “unattached” had at least one kinetochore signal away from and off-axis from the spindle, even though the main kinetochore mass was still bilobed. Cells were classified as “unattached and declustered” if there was at least one unattached kinetochore and the main kinetochore mass was also declustered. Data are from two independent technical trials of two biological replicates. In each replicate, n = 50 cells were counted. Graphs are of the mean and standard error of the mean (GraphPad Prism).

## Reagents

**Table:** 

Strain Number	Genotype
DDY 5818	*MAT a, cdc23-1, SPC105-3x flexible linker-yoEGFP::KanMX, TUB1::PHIS3-mRuby2-TUB1::HPH, his3-∆200, leu2-3,112, lys2-801(am), ura3-52*
DDY 5820	*MAT a, cdc23-1, SPC105-3x flexible linker-yoEGFP::KanMX, kip3∆::HIS3MX6, TUB1::PHIS3-mRuby2-TUB1::HPH, his3-∆200, leu2-3,112, lys2-801(am), ura3-52*
DDY 5965	*MAT alpha, cdc23-1, STU2-3V5-AID::CaURA3, SPC105-3x flexible linker-yoEGFP::KanMX, TUB1::PHIS3-mRuby2-TUB1::HPH, OsTIR1::LEU2, his3-∆200, leu2-3,112, lys2-801(am), ura3-52*
DDY 5966	*MAT alpha, cdc23-1, STU2-3V5-AID::CaURA3, SPC105-3x flexible linker-yoEGFP::KanMX, kip3∆::HIS3MX6, TUB1::PHIS3-mRuby2-TUB1::HPH, OsTIR1::LEU2, his3-∆200, leu2-3,112, lys2-801(am), ura3-52*
DDY 5967	*MAT alpha, cdc23-1, KIP3-EGFP:KanMX, mRuby2-TUB1::HYG, his3∆200, lys2-801 am, ura3-52, leu2-3,112*
DDY 5968	*MAT alpha, cdc23-1, STU2-EGFP:KanMX, SPC105-mScarlet-I::LEU2, ura3-52::PHIS3-HaloTag-TUB1::URA3, his3-∆200, leu2-3,112, lys2-801*
DDY 5969	*MAT alpha, cdc23-1, STU2-3V5-AID::CaURA3, SPC105-mScarlet-I::LEU2, KIP3-yoEGFP::KanMX, pGPD1-OsTIR1::HIS3, his3-∆200, leu2-3,112, lys2-801(am), ura3-52*
DDY 5970	*MAT alpha, cdc23-1, SPC105-3x flexible linker-yoEGFP::KanMX, KIP3-HaloTag::kanMX6, TUB1::PHIS3-mRuby2-TUB1::HPH, his3-∆200, leu2-3,112, lys2-801, ura3-52*
